# Genome-Wide Linkage, Exome Sequencing and Functional Analyses Identify *ABCB6* as the Pathogenic Gene of Dyschromatosis Universalis Hereditaria

**DOI:** 10.1371/journal.pone.0087250

**Published:** 2014-02-03

**Authors:** Hong Liu, Yi Li, Ken Kwok Hon Hung, Na Wang, Chuan Wang, Xuechao Chen, Donglai Sheng, Xi’an Fu, Kelvin See, Jia Nee Foo, Huiqi Low, Herty Liany, Ishak Darryl Irwan, Jian Liu, Baoqi Yang, Mingfei Chen, Yongxiang Yu, Gongqi Yu, Guiye Niu, Jiabao You, Yan Zhou, Shanshan Ma, Ting Wang, Xiaoxiao Yan, Boon Kee Goh, John E. A. Common, Birgitte E. Lane, Yonghu Sun, Guizhi Zhou, Xianmei Lu, Zhenhua Wang, Hongqing Tian, Yuanhua Cao, Shumin Chen, Qiji Liu, Jianjun Liu, Furen Zhang

**Affiliations:** 1 Shandong Provincial Institute of Dermatology and Venereology, Provincial Academy of Medical Science, Jinan, Shandong, China; 2 Shandong Provincial Hospital for Skin Diseases, Shandong University, Jinan, Shandong, China; 3 Shandong Provincial Key Lab for Dermatovenereology, Jinan, Shandong, China; 4 Shandong Provincial Medical Center for Dermatovenereology, Jinan, Shandong, China; 5 Human Genetics, Genome Institute of Singapore, A*STAR, Singapore, Singapore; 6 Shandong provincial Eye Hospital, Jinan, Shandong, China; 7 National Skin Centre, Singapore, Singapore; 8 Institute of Medical Biology, A*STAR, Singapore, Singapore; 9 Weifang People’s Hospital, Weifang, Shandong, China; 10 Institute of Dermatology, Chinese Academy of Medical Sciences and Peking Union Medical College Nanjing, Jiangsu, China; 11 Shandong University, Jinan, Shandong, China; 12 School of Life Sciences, Anhui Medical University, Hefei, Anhui, China; 13 Shandong Clinical College, Anhui Medical University, Jinan, Shandong, China; Oslo University Hospital, Norway

## Abstract

**Background:**

As a genetic disorder of abnormal pigmentation, the molecular basis of dyschromatosis universalis hereditaria (DUH) had remained unclear until recently when ABCB6 was reported as a causative gene of DUH.

**Methodology:**

We performed genome-wide linkage scan using Illumina Human 660W-Quad BeadChip and exome sequencing analyses using Agilent SureSelect Human All Exon Kits in a multiplex Chinese DUH family to identify the pathogenic mutations and verified the candidate mutations using Sanger sequencing. Quantitative RT-PCR and Immunohistochemistry was performed to verify the expression of the pathogenic gene, Zebrafish was also used to confirm the functional role of ABCB6 in melanocytes and pigmentation.

**Results:**

Genome-wide linkage (assuming autosomal dominant inheritance mode) and exome sequencing analyses identified *ABCB6* as the disease candidate gene by discovering a coding mutation (c.1358C>T; p.Ala453Val) that co-segregates with the disease phenotype. Further mutation analysis of *ABCB6* in four other DUH families and two sporadic cases by Sanger sequencing confirmed the mutation (c.1358C>T; p.Ala453Val) and discovered a second, co-segregating coding mutation (c.964A>C; p.Ser322Lys) in one of the four families. Both mutations were heterozygous in DUH patients and not present in the 1000 Genome Project and dbSNP database as well as 1,516 unrelated Chinese healthy controls. Expression analysis in human skin and mutagenesis interrogation in zebrafish confirmed the functional role of *ABCB6* in melanocytes and pigmentation. Given the involvement of *ABCB6* mutations in coloboma, we performed ophthalmological examination of the DUH carriers of *ABCB6* mutations and found ocular abnormalities in them.

**Conclusion:**

Our study has advanced our understanding of DUH pathogenesis and revealed the shared pathological mechanism between pigmentary DUH and ocular coloboma.

## Introduction

Dyschromatosis universalis hereditaria (DUH) is a rare Mendelian disease, characterized by asymptomatic hyper- and hypo-pigmented macules in variable distributions and patterns, which was initially described by Ichikawa and Hiraga in 1933 [1]. Most DUH patients do not show other symptoms associated with the typical skin abnormalities, although high tone deafness, ocular abnormalities, photosensitivity, neurosensory hearing defects, learning difficulties, mental retardation, epilepsy, insulin-dependent diabetes mellitus, erythrocyte, platelet and tryptophan metabolism abnormalities, and small stature had been found occasionally [2].

As an autosomal dominant (OMIM 127500) or recessive disorder (OMIM 612715), the genetic loci of DUH have been mapped to chromosome 6q24.2-q25.2 [3] and 12q21-q23 [4] by the linkage analysis, but the molecular basis of DUH had remained unknown until recently when Zhang’s group reported ABCB6 as a causative gene of DUH [5]. We performed linkage and exome sequencing analyses in a Chinese DUH pedigree and identified ABCB6 as the pathogenic gene of DUH almost at the same time as Zhang’s group. The expression of ABCB6 were demonstrated in human skin tissue and melanocytes and the knockdown of ABCB6 expression was shown to cause the reduction of the number of mature melanocytes in zebrafish, which provided solid and convincing evidences for ABCB6 to be a disease gene for DUH.

## Results

### Genome-wide Linkage Analysis to Identify the Causal Regions

**Figure 1:**
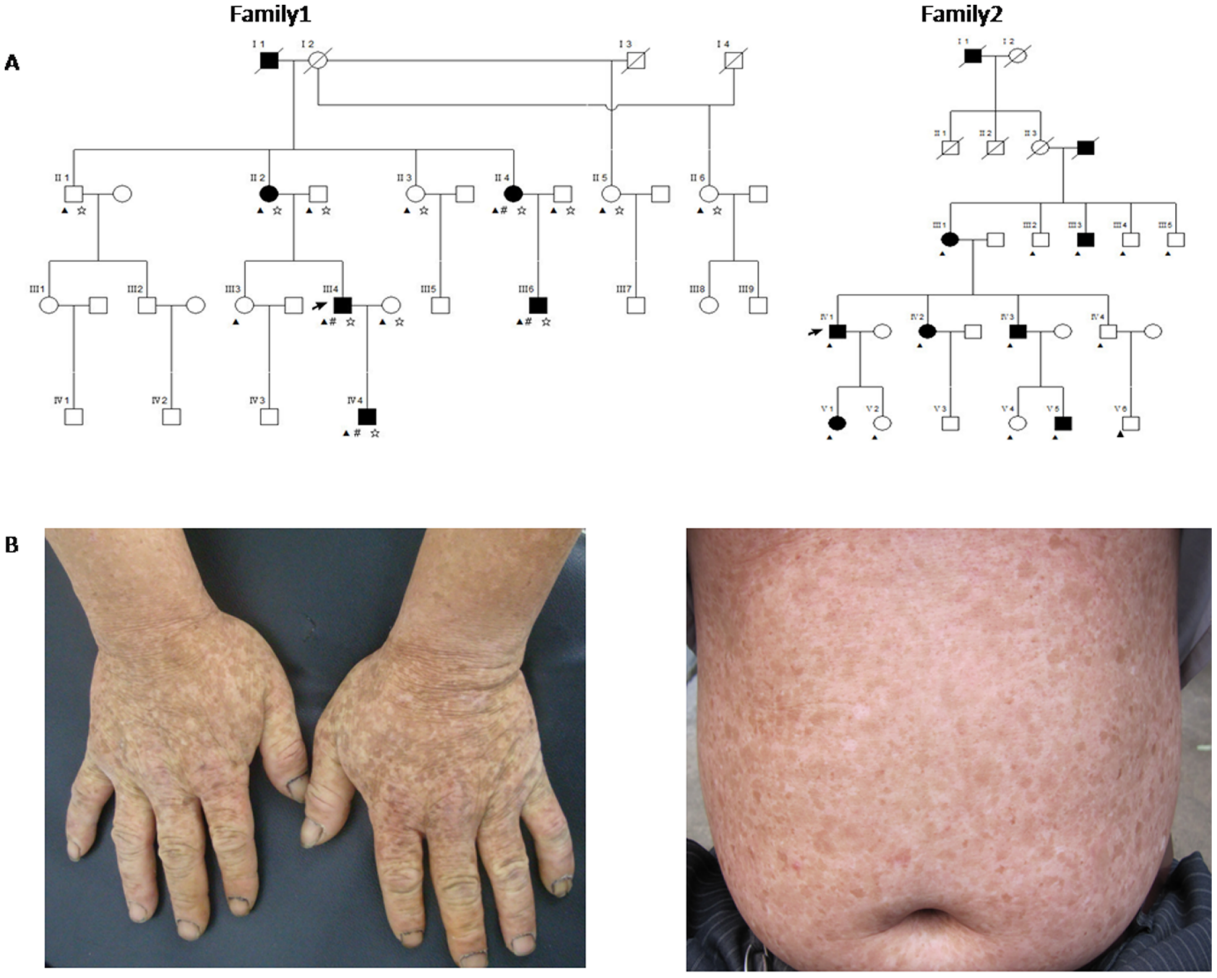
Family trees and clinical manifestations. **A** Shown are the pedigree of family 1 and 2 with autosomal dominant DUH. “#”represents the individuals used in exome sequencing analysis, “☆”represents the individuals used in the linkage analysis; “▴”represents the individuals subjected to Sanger sequencing analysis. **B**: Clinical manifestations of DUH patients, both hands and abdomen with hyper- and hypo-pigmented macules in variable distribution and patterns.

Here, we performed the genome-wide linkage and exome sequencing analyses in a multi-generational DUH family of Chinese ethnicity. First, we genotyped the 12 individuals of the Family (Family 1) using Illumina Human 660W-Quad BeadChip **(** **Figure 1A** **).** Multipoint parametric linkage analysis was performed in Merlin [6] by using pruned autosomal SNPs (with LD r^2^<0.1 in Chinese population data) and assuming a dominant inheritance of disease phenotype. Maximal LOD score of 1.81 was obtained on chromosomes 2q34-2q37, 10q25, 13q12-13q14, 17q25 and 20p12 **(Figure S1 in Text S1)**. Co-segregation of haplotypes within the 5 critical regions identified by linkage, which spanned from 4 MB to 24 MB in size, was confirmed by haplotype analysis using Haplopainter [7].

### Exome Sequencing Analysis to Explore ABCB6 as the Pathogenic Gene

We then performed exome sequencing analysis in four affected individuals (II4, III4, III6 and IV4) of the Family 1 **(** **Figure 1** **A)**. An average of 8.76 billion bases of sequences was generated for each individual. BWA was invoked to map the reads to hg19 human reference genome [8]. On average, each sample had a mean coverage of 39.08 and 86.4% of the exome sequences were covered at 10X or more **(Table S1 in Text S1)**. GATK Unified Genotyper with the recommended filtering criteria was invoked to call single nucleotide variants (SNV) and indels [9]. For each individual, 17,579 coding variants were identified on average; the percentage of novel SNVs (SNPs not in dbSNP132) was 6.5%; Ti/Tv ratios for novel SNVs and SNVs in dbSNP132 were 2.55 and 3.22 respectively. When considering only non-synonymous and splice site SNVs, 4,625 were shared by all the four patients; and 98 of which were not in 1000 Genome Project (Phase I, Oct 2011) or dbSNP 132. Only one SNV, chr2∶220079139, heterozygous in four affected individuals, lied within the critical linkage regions defined by haplotype co-segregation analysis. This coding mutation (c.1358C>T; p.Ala453Val) is in the exon 7 of *ABCB6*, and was predicted to be damaging by SIFT and probably damaging by PolyphenII. The mutation was not found in the 500 exomes of Chinese origin.

### Sanger Sequencing to Verify the Candidate Gene ABCB6

**Figure 2:**
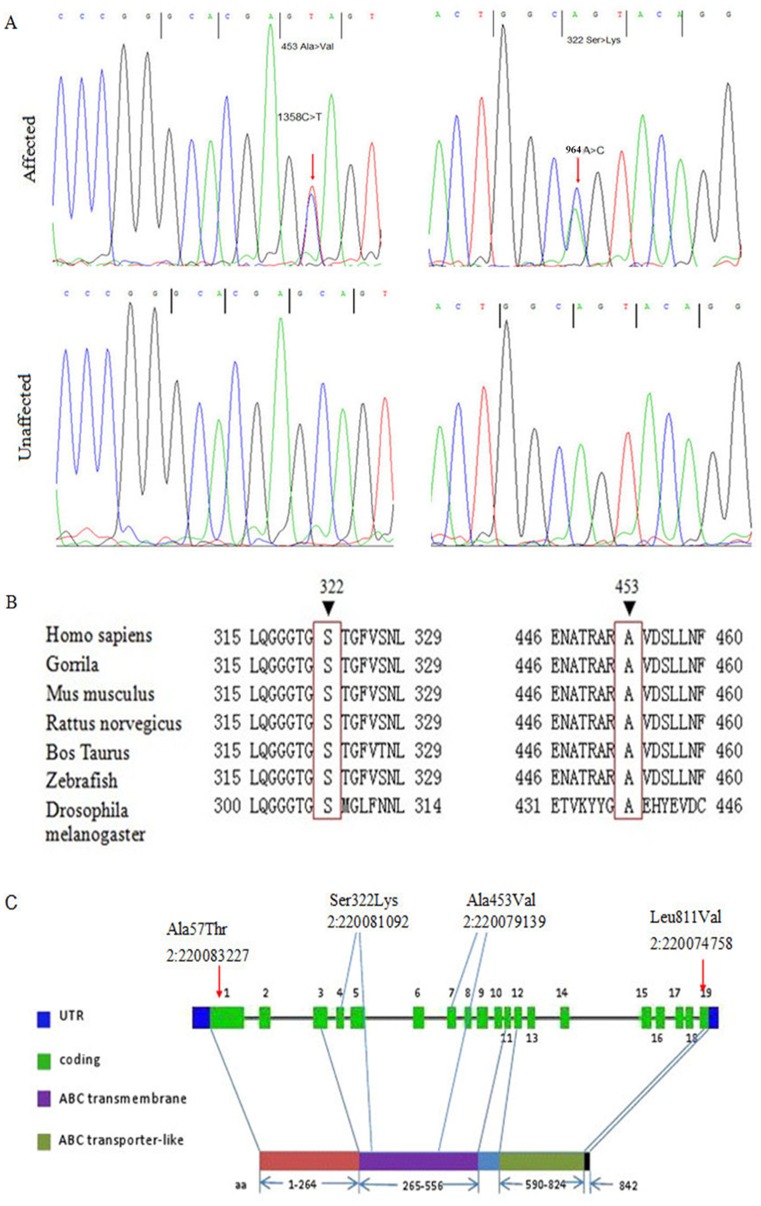
Mutations in ABCB6. **A:** Two mutations in *ABCB6* and their sequencing traces, including c.1358C>T; p.Ala453Val in the Family 1 and c.964A>C; p.Ser322Lys in the Family 2; Arrows indicate the location of the two mutations. **B:** A partial sequence of ABCB6 was compared with other species’ orthologs. Arrows indicate the location of the two mutations identified in patients with DUH. **C:** *ABCB6* exon structure, where c.964A>C; p.S322L and c.1358C>T; p.A453V are DUH mutations identified in this paper, c.169G>A; p.A57T and c.2431C>G; p.L811V are coloboma mutations^7^.

To verify the mutation (c.1358C>T; p.Ala453Val), 13 individuals of the Family 1 **(** **Figure 1A** **)** were Sanger sequenced for all the exons of *ABCB6*. The mutation was heterozygous in all the five affected individuals and absent in all the eight unaffected individuals **(** **Figure 2A** **)**, and no other mutations were found within this gene. 1,016 ethnicity-matched healthy controls were also Sanger sequenced for the exon 7 of *ABCB6*, and none of them carried the mutation.

We further sequenced all the exons and exon-intron boundaries of *ABCB6* in other four DUH pedigrees and two sporadic cases of Chinese ethnicity by Sanger sequencing. A new coding mutation in exon 4 (chr2∶220081092, c.964A>C; p.Ser322Lys) was discovered in Family 2, which was heterozygous in all the seven affected individuals and absent in all the seven unaffected members **(** **Figure 2A** **)**. The single-point linkage LOD score based on Sanger sequence data for family 2 is 3.08; and the single-point HLOD score for family 1 and 2 is 5.08 (corresponding p-value = 6.6×10^−7^). This mutation was predicted to be tolerated and possibly damaging by SIFT and PolyphenII respectively. Similarly, the mutation was not presented in the 500 exomes and 1,016 healthy controls of Chinese origin (data not shown). No mutations of *ABCB6* were identified in the other three families (**Figure S2 in Text S1**) and two sporadic cases that are absent from the 1000 Genome Project and dbSNP datasets and co-segregate with disease phenotype, indicating the genetic heterogeneity of DUH as suggested by previous linkage studies[3]–[4].

### ABCB6 Expression Analysis

To confirm the functional role of *ABCB6* in pigmentation, we first investigated the expression of *ABCB6* in the skin tissues of four DUH carries of *ABCB6* mutations (III4,II4,IV4 from family 1 and IV3 from family 2) and four unrelated health controls using quantitative RT-PCR. Expression of *ABCB6* mRNA was detected in both DUH affected individuals and healthy controls, but no significant difference was found between the affected and unaffected individuals (P = 0.44) **(Figure S3 in Text S1)**. We then performed immunohistochemistry (IHC) analysis in human primary melanocyte cell line (HEM cell) and the formalin-fixed paraffin-embedded (FFPE) skin tissue of one affected individual (III4 from family 1) and one healthy control. The expression of ABCB6 protein was clearly detected in the HEM cell (**Figure S4A in Text S1**). In human skin, ABCB6 could be detected in melanocytes, but not keratinocytes of the basal layer in both the affected and healthy control individuals (**Figure S4C and E in Text S1**). Similarly, no significant expression difference was found between the patient and healthy control. The lack of the expression difference of ABCB6 in skin tissue between the patients and healthy controls is not surprising, because both the mutations identified here are missense mutations within the trans-membrane domain of ABCB6 that more likely influence the subcellular location of ABCB6 instead of its expression level, and thus cause a dominant negative effect on the development of disease phenotype.

### Morpholino Knockdown of ABCB6 in Zebrafish and Rescue Studies

**Figure 3:**
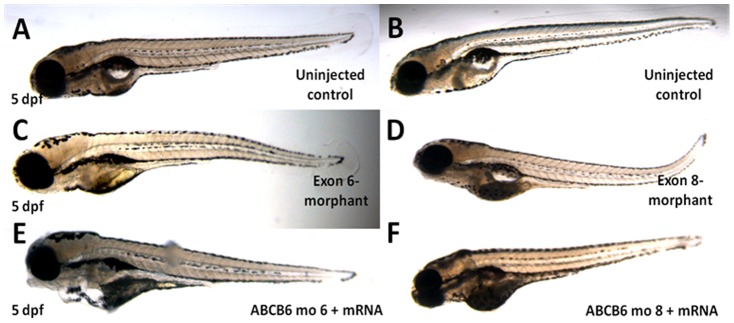
Phenotypes of the microinjected zebrafish. Morpholinos against exon 6 and exon 8 of *zABCB6* and wildtype *zABCB6* mRNA transcript (in case of rescue) were designed and injected into one- to two-cell stage embryos. No obvious phenotypic changes were observed in the morphants (C and D) and rescued (E and F) embryos after 5 days (5pdf) with reference to their uninjected counterparts (A and B).

**Figure 4:**
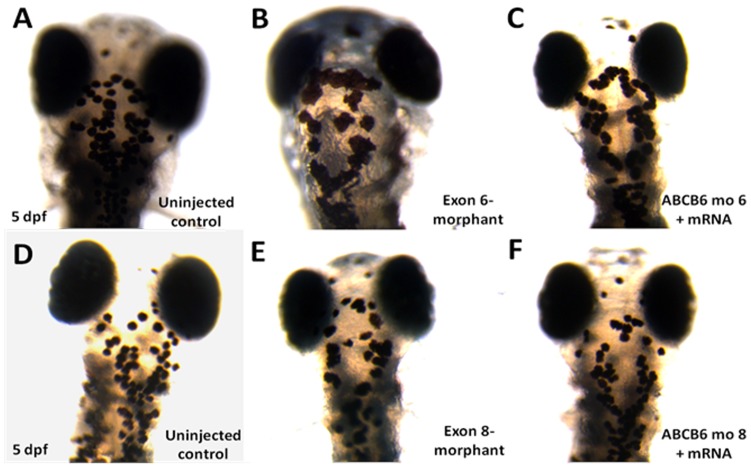
Number of mature melanocytes in the head region in the morphants and rescued embryos (5 dpf). Immobilized embryos were dorsally oriented and the number of mature melanocytes was counted in the defined region of the head. Reduction in melanocyte number was observed in both exon 6- and exon 8-morphants (B and E). Co-injection of wildtype *hABCB6* mRNA transcript significantly rescued the loss of melanocyte phenotype by increasing the number of mature melanocytes (C and F).

**Figure 5:**
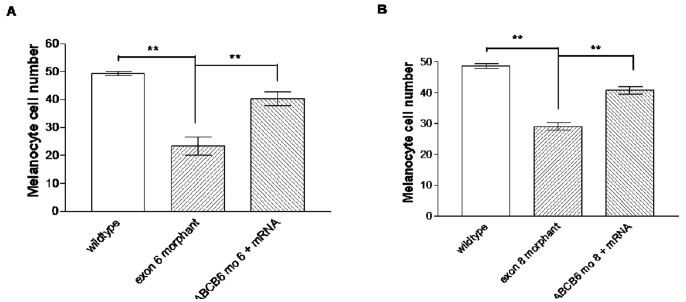
Number of melanocytes in the defined head regions in uninjected control (wildtype), exon 6- and exon 8-morphants and rescued embryos. **P<0.001 (ANOVA one-way analysis of variance with Tukey’s multiple comparison post test).

We also carried out further functional investigation of *ABCB6* in zebrafish. We first performed a knockdown analysis of zebrafish *ABCB6* homolog (*zABCB6*) by using morpholinos targeting the exon 6 and exon 8 of *zABCB6* (which are homologous to the exon 4 and exon 7 of human *ABCB6*) (**Figure S5 in Text S1**). No morphological abnormalities were found in surviving embryos at 5 dpf **(** **Figure 3** **)**. In developing zebrafish embryos, melanocytes can be first visualized at 25 hpf and then undergo differentiation to become “spot-like” mature melanocytes at 5 dpf (**Figure 4A and D**). By counting the number of pigmented melanocytes present within the head region at 5 dpf, we observed a significant higher number of mature melanocytes in the uninjected control embryos (48.84±0.68, n = 57) than the injected embryos with the exon 6-morphants (23.38±3.20, n = 13, p<0.001) (**Figure 4B**) and the ones with the exon 8-morphants (29.04±1.18, n = 24, p<0.001) (**Figure 4E**). To confirm that the mutant phenotype of the reduction of mature melanocytes in *ABCB6* morphants is due to the specific effect of the ABCB6 expression knockdown, we co-injected *ABCB6* morpholinos with full length hABCB6 mRNA transcript and found that the co-injection of full length mRNA (with morpholinos) was able to rescue, at least partially, the mutant phenotype in *ABCB6* morphants. Co-injected embryos showed significant higher number of mature melanocytes (exon 6-morphant-rescue: 39.71±2.47, n = 19, p<0.001; exon 8-morphant-rescue: 40.77±1.26, n = 30, p<0.001) (**Figure 4C and F**) than the morphants (**Figure 5A and B**). Our functional investigation of *ABCB6* in zebrafish confirmed the involvement of *ABCB6* in pigmentation by influencing the number of mature melanocytes, providing the critical functional evidence for the role of *ABCB6* in the pathogenesis of DUH.

## Discussion

A recent publication showed that two missense mutations of *ABCB6* (c.2431C>G; p.Leu811Val and c.169G>A; p.Ala57Thr) caused ocular coloboma [10]. And also the co-exist of pigmentation and ocular abnormalities were reported to be observed in some cases [2]. This prompted us to carry out careful ophthalmological examination of the DUH patient carriers of *ABCB6* mutations. Although no microphthalmia and anophthalmia were observed in the affected individuals, the detailed clinical examination, visual-acuity testing and ocular imaging revealed that II5 and III4 of the Family 1 had abnormal pitting lack of iris, and IV4 of the Family 1 had high intra-ocular pressure (L:24 mmHg; R:19 mmHg) (**Figure S6 in Text S1**). However, no abnormal eye manifestations were found in the IV1 of the Family 2.

Although both Wang et al’s study [10] and ours used zebrafish as a model to investigate the functions of ABCB6, experimental design is different between two studies. The morpholino in Wang et al targeted the ATG start codon and exon2-intron2 boundary, with the former aiming to block the translation of the whole protein, whereas our morpholinos targeted at exon 6 or exon 8 aiming to the deletion of specific domains of the protein. Analyzing the early stages of development at 36 hpf, Wang et al reported significant delay in development and coloboma-related eye defects in their morphants. Our experiments focused on late stage of development 5 dpf when mature melanocytes are fully differentiated in the zebrafish and found less pigmentation in the morphants. Although further studies are needed to confirm, these results may suggest differential functions of ABCB6 at different stages of development.

It is very intriguing that the different mutations of *ABCB6* can cause diverse disease phenotypes, pigmentary DUH and ocular coloboma. The two DUH mutations lie in the ABC transmembrane domain, whereas one coloboma mutation (c.2431C>G; p.Leu811Val) located in the transporter-like domain, and the other one (c.169G>A; p.Ala57Thr) did not lie in any established domain **(** **Figure 2** **C)**. This may suggest that ABCB6 protein has diverse biological functions, and mutations affecting its different domains may thus cause diverse disease phenotypes.

*ABCB6*, located at 2q36, contains 19 exons and is a member of the superfamily of ATP-binding cassette (ABC) transporters, which transport various molecules across extra- and intra-cellular membranes. *ABCB6* is expressed ubiquitously, including in the skin. There are two conserved domains in the protein, namely ABC transmembrane domain and ABC transporter-like domain **(** **Figure 2C** **)**. Sequence comparison of *ABCB6* across different species showed that both the amino acids affected by DUH mutations (c.1358c>T;p.Ala453Val and c.964A>C; p.Ser322Lys) are highly conserved (**Figure 2B**), implying that these two residues are key to normal biological function of ABCB6. ABCB6 plays an important role in hemo-synthesis and multi-drug resistance as the porphyrin transporter [11]–[13]. It also functions as a glycosylated protein targeted to intracellular vesicular membranes and plays a critical role in cellular transition and metal homeostasis, especially copper homeostasis [14]. Copper is thought to be particularly important for vertebrate pigmentation as a cofactor for the enzyme tyrosinase that is essential to catalyze the initial steps of melanin synthesis within the melanosome [15]–[16]. While still being speculative, it is possible that the DUH mutations of *ABCB6* may result in abnormal copper homeostasis, which influence the activity of tyrosinase and further lead to the abnormal melanin synthesis. Further studies are needed to investigate the molecular functions of the different domains of ABCB6 and the mechanisms underlying the involvement of *ABCB6* mutations in diverse disease phenotypes. Although the genetic methods we used for disease gene discovery are similar to Zhang’s group [5], the functional validation of the identified disease gene is different between the two studies. In our study, zebrafish model was established to investigate the role of ABCB6 in pigmentation; while in theirs, subcelluar localization experiment was performed to detect the location of wild-type ABCB6 in a B16 mouse melanoma cells, and to show that disease-causing mutations of ABCB6 resulted in its retention in the Golgi apparatus. The two studies supported each other in some aspect. In addition, we found the co-existence of pigmentation and ocular abnormalities in our DUH patients, which provides critical evidence for the diverse role of ABCB6 in both pigmentation and ocular development.

In summary, we have discovered *ABCB6* as a disease gene for DUH with solid genetic and functional evidences through genome-wide linkage, exome sequencing, and functional investigation in human tissues and zebrafish model. Our discovery has expanded the novel disease-causing mutations of ABCB6 to five, advanced our understanding of DUH pathogenesis and revealed the shared pathogenic mechanism between pigmentary DUH and ocular coloboma.

## Materials and Methods

### Subject

We recruited five unrelated DUH families (four in China and one in Singapore, totalling 23 affected individuals and 16 unaffected family members), two sporadic case (one in China and one in Singapore, totalling two affected individuals and two unaffected family members) of Chinese ethnicity in our study. In addition, the dataset of 500 exomes and additional 1,016 unrelated normal controls of Chinese ethnicity were also analyzed in our study. After obtaining informed consent from all participants, all the family members were carefully examined by at least two experienced dermatologists, and the clinical diagnosis of DUH in all the cases is based on the generalized and random distribution of small hypo- and hyper-pigmented lesions.

EDTA anticoagulated venous blood samples were collected from all participants. Genomic DNA was extracted from peripheral blood lymphocytes by standard procedures using FlexiGene DNA kits (Qiagen). Total RNA was extracted from skin biopsies of 4 DUH patients(III4, II4, IV4 from family 1, IV3 from family 2) and 4 health controls using TRIzol (Invitrogen Life Technologies).

The study was approved by the institutional review board at Shandong Provincial Institute of Dermatology and Venereology and the National Health Group, Domain Specific Review Board for National Skin Centre, Singapore. Written informed consent was obtained from all participants, or their guardian. All experiments of zebra fish were approved by and conducted in accordance with the guidelines established by the Institutional Animal Care and Use Committee(IACUC) at the Institute of Medical Biology, A*STAR, Singapore.

### Genome-wide Linkage Analysis

For the genome-wide linkage analysis, approximately 200 ng of genomic DNA of the 12 individuals from the Family 1 was used for the genotyping analysis by using Illumina Human 660W-Quad BeadChip. Briefly, each sample was whole-genome amplified, fragmented, precipitated and resuspended in appropriate hybridization buffer. Denatured samples were hybridized on prepared Illumina Human 660-Quad BeadChip. After hybridization, the BeadChip oligonucleotides were extended d by a single labeled base, which was detected by fluorescence imaging with an Illumina Bead Array Reader. Normalized bead intensity data obtained for each sample were loaded into the Illumina GenomStudio software, which converted fluorescence in tensities into SNP genotypes.

After quality control filtering, 589,209 SNPs were remained for linkage analysis. Familial relationship check based on IBD sharing was carried out to confirm the collected pedigree information. Multipoint parametric linkage analysis was performed in Merlin by using the pruned autosomal SNPs (with LD <0.1 in population data) and assuming dominant inheritance with a disease allele frequency of 0.001, penetrance rate of 0.99 and phenocopy rate of 0.01. The linkage critical regions were determined by haplotype co-segregation analysis using Haplopainter.

### Exome Capture, Sequencing and Variant Detection

We performed the exome capture using Agilent SureSelect Human All Exon Kits according to the manufacturer’s protocols. Genomic DNA samples with high quality were sheared and randomly fragmented using the Covaris instrument with a base pair peak of 150–200 bp. The sheared DNA samples were purified using the Agencourt AMPure XP beads and tested with the Agilent 2100 Bioanalyzer. The ends were repaired and the 3′ end of the fragments was added ‘A’ bases. Adapters were then ligated to both ends of the fragments. The adaptor-ligated templates were further purified using Agencourt AMPure SPRI beads and fragments with insert size 250 bp were excised. The adaptor-ligated library was amplified by ligation-mediated PCR, purified and hybridized to the SureSelect Biotinylated RNA Library (BAITS) for enrichment. Hybridized fragments were bound to the strepavidin beads, whereas non-hybridized fragments were washed out. The captured library was amplified, purified and then were tested using the Agilent 2100 Bioanalyzer to estimate the magnitude of enrichment.

Each captured library was then loaded on a HiSeq 2000 platform, and paired-end sequencing was performed with read lengths of 100 bp, which provided 50× average coverage depth for each sample. Raw image files were processed by Illumina base-calling Software 1.7 for base calling with default parameters.

Sequence reads from each individual were aligned to the human reference genome (hg19) using BWA(Burrows-Wheeler Alignment) version 0.5.9 rcl with default parameters. Reads that had duplicated start sites were removed; the remaining reads that mapped on or near the target were collected for subsequent analyses and variant calling. The consensus genotypes, SNP and indel variants in the target regions were called by Genome Analysis Toolkit v1.0.5974 with the recommended parameters. The filtering criteria were based on the “best practice variant detection with the GATKv3”, that is “QD <2.0”, “MQ <40.0”, “FS >60.0”, “HaplotypeScore >13.0”, “MQRankSum<−12.5”, “ReadPosRankSum<−8.0” for SNV, and “QD <2.0”, “ReadPosRankSum<−20.0”, “InbreedingCoeff<−0.8”, “FS >200.0” for indel.

### Sanger Sequencing

Sanger sequencing was performed to confirm the variants identified by exome sequencing and search for additional mutations. All the 19 exons of *ABCB6* gene and their flanking intronic sequences of 200 bps were amplified by PCR. The primer sequences were designed by Primer3(v0.4.0). After amplification, products were purified and sequenced on DNA sequencing system (model 3130XL; ABI). Mutations were identified by comparing with the reported DNA reference sequence (GenBank accession number: NG_032110). All the identified mutations were verified by the subsequent two-direction sequencing. Both the patients and control samples were analyzed using the same protocol.

### Quantitative RT-PCR

Total RNA was extracted from the skin biopsies of four DUH patients(III4,II4,IV4 from family1, IV3 from family 2) and four health controls using TRIzol (Invitrogen Life Technologies). Quantitative RT-PCR was performed using a 7300 Real Time PCR System (Applied Biosystems) and manufacturer protocols. GAPDH was used as an internal control, and the specific products of human ABCB6 and GAPDH were implicated using the following primers: ABCB6: forward: 5′-CAGCAGGGACAGGAAGAA-3′; reverse: 5′-CCAAGACCAGGATGAAAT-3′, GAPDH: forward: 5′-ACCACAGTCCATGCCATCAC-3′; reverse: 5′-TCCACCACCCTGTTGCTGTA-3′. The threshold cycle (CT) values were used to show the expression level of the target and reference genes and determine the relative mRNA levels that were expressed as the fold change of the target gene relative to the reference gene. Error bars represent the standard errors for three independent experiments.

### Immunohistochemistry

Skin biopsies were obtained from DUH patient (III4 from family 1) and healthy control. All skin biopsies were fixed with 10% neutral buffered formalin, and then subsequently embedded in paraffin. 4-µm sections of tissue blocks were microtome sectioned, mounted on glass slides and dried at 48°C for 4 h, then the sections were deparaffinized in xylene and rehydrated with a series of graded ethanol. The antigen retrieval method for tissue sections is Heat Induced Epitope Retrieval (HIER) that was performed by heating the slides immersed in retrieval buffer, pH 6.0, for 4 minutes at 125°C in the pressure boiler. Next, the sections were immersed in potassium permanganate solution for 1 minute to removed pigmentations will TritonX-100 solution. Human primary melanocyte cell line HEM was maintained in MelM medium (Scien Cell) supplemented with 0.25% FBS (Scien Cell), penicillin, and streptomycin. The cells were incubated on round coverslip for 12 h, and washed with PBS after the serum was removed. Then, the cells were fixed on the coverslip with 4% paraformaldehyde solution and permeabilized in 0.5%TritonX-100 solution.

To inactivate endogenous peroxidase activity, the sections and cells were treated with 3% H_2_O_2_ in PBS for 10 minutes. For immunostaining, a primary rabbit polyclonal ABCB6 antibody (HPA046723, Sigma-aldrich) was employed in 1∶15 dilution with PBS and incubated for 3 h at 37°C in a humid chamber. After several subsequent washing steps with PBS for at least 15 min, the sections and cells were incubated at 37°C for 30 minutes using the goat anti-rabbit IgG (H+L)-HRP Secondly antibody. Visualization was achieved using 3′3-diaminobenzidine tetra hydrochloride substrate (DAB). The sections and cells were counterstained with hematoxylin, dehydrated with a series of graded ethanol and paraffinized in xylene.

### Zebrafish Husbandry

Zebrafish were maintained according to methods described in The Zebrafish Book [17] under the ordinance from the Biological Resource Centre, Agency for Science, Technology and Research (A*STAR), for the responsible care and the use of laboratory animals. The fishes were housed in the Danio Unit, Institute of Molecular and Cellular Biology (IMCB) and embryos were collected by breeding wild-type strains AB line.

### Morpholinos Against ABCB6

Four different morpholino antisense oligonucleotides against zABCB6 (intron 6 splice donor junction, EI-TTGTTGTCTCTGACCTGAGCAGGCT; intron 6 splice acceptor junction, IE-GACGCACCTGCACATGGAGACAGAT; intron 8 splice donor junction, EI-CAAACAAAGAGAAGCCCTACCGTCT and intron 8 splice acceptor junction, IE-AGAGCTGTCGGAGACACGGATAAAA) were obtained from Gene Tools (Philomath, OR) and injected at the one- to two-cell stage at the concentrations of 125 µM for exon 6 knockdown and 62.5 µM for exon 8 knockdown. Extent of knockdown achieved by morpholinos was evaluated using RT-PCR with primer pairs (ABCB6_F1: GCCATGACGGTGTGTGTTTAT and ABCB6_R1: AAAGACTATAAGGCCAAACCAGG) and (ABCB6_F2: TCAGCATCTTCCCCACCA and ABCB6_R2: TCAGCAAACCCAAACCAATG).

### Rescue Experiments

Capped hABCB6 poly(A)-tailed mRNA transcript was in vitro transcribed from linearized hABCB6-pcDNA3.1/myc-His B plasmid according to the protocol in the mMESSAGE mMACHINE T7 Ultra kit (Life Technologies, CA). Rescue experiment was performed by co-injecting the splice-blocking morpholinos and the full length zABCB6 mRNA transcript at a concentration of 200 ng into one- to two-cell stage zebrafish embryos.

### Imaging and Melanocyte Counting

Five day post fertilization (5 dpf) zebrafish were assessed for phenotypic changes in distribution and number of melanocytes. They were first exposed to light for at least half an hour to contract the melanocytes. Afterwards, they were fixed in 4% paraformaldehyde for overnight and oriented dorsally in 1% low melting agarose and imaged with an upright stereomicroscope (Leica microsystems). The number of melanocytes was counted in the captured images within a defined head region [18]–[19].

## Supporting Information

Text S1 **Combined supporting information file containing Figures S1–S6 and Table S1.** Figure S1: The pedigree of four families with autosomal dominant DUH. (DOC). Figure S2: LOD score of parametric linkage analysis vs genetic map for 22 chromosomes. (DOC). Figure S3: The expression of ABCB6 mRNA in DUH patients and health controls. (DOC). Figure S4: The protein level of ABCB6 in normal melanocyte and skin biopsies. (DOC). Figure S5: Sequence homology of ABCB6 between species. (DOC). Figure S6: Number of melanocytes in the defined head regions of zebrafish embryos. (DOC). Table S1: Information for exome sequencing reads. (DOC).
(DOCX)
