# Supplementary material for: Genome-Wide Linkage, Exome Sequencing and Functional Analyses Identify ABCB6 as the Pathogenic Gene of Dyschromatosis Universalis Hereditaria
Source: PLoS One. 2014 Feb 3;9(2):e87250. doi: 10.1371/journal.pone.0087250 (PMC3911924; doi:10.1371/journal.pone.0087250)
Supplement: Text S1 — Combined supporting information file containing Figures S1–S6 and Table S1. Figure S1: The pedigree of four families with autosomal dominant DUH. (DOC). Figure S2: LOD score of parametric linkage analysis vs genetic map for 22 chromosomes. (DOC). Figure S3: The expression of ABCB6 mRNA in DUH patients and health controls. (DOC). Figure S4: The protein level of ABCB6 in normal melanocyte and skin biopsies. (DOC). Figure S5: Sequence homology of ABCB6 between species. (DOC). Figure S6: Number of melanocytes in the defined head regions of zebrafish embryos. (DOC). Table S1: Information for exome sequencing reads. (DOC). (DOCX) [file pone.0087250.s001.docx]

Genome-wide linkage, exome sequencing and functional analyses identify ABCB6 as the pathogenic gene of

dyschromatosis universalis hereditaria

**Supplementary Figure 1** shown the pedigree of four families with autosomal dominant DUH. “#”represents the individuals used in exome sequencing analysis, ☆”represents the individuals used in the linkage analysis; “▲”represents the individuals subjected to Sanger sequencing analysis.


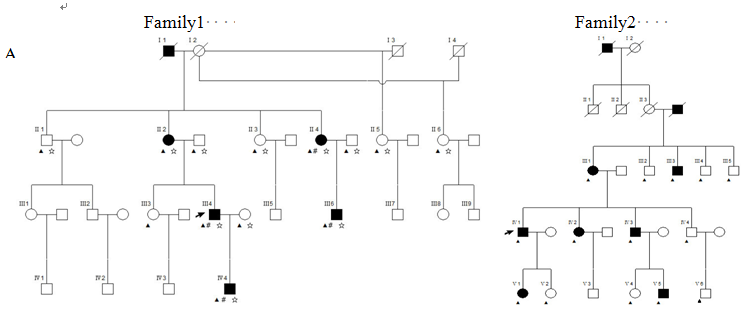


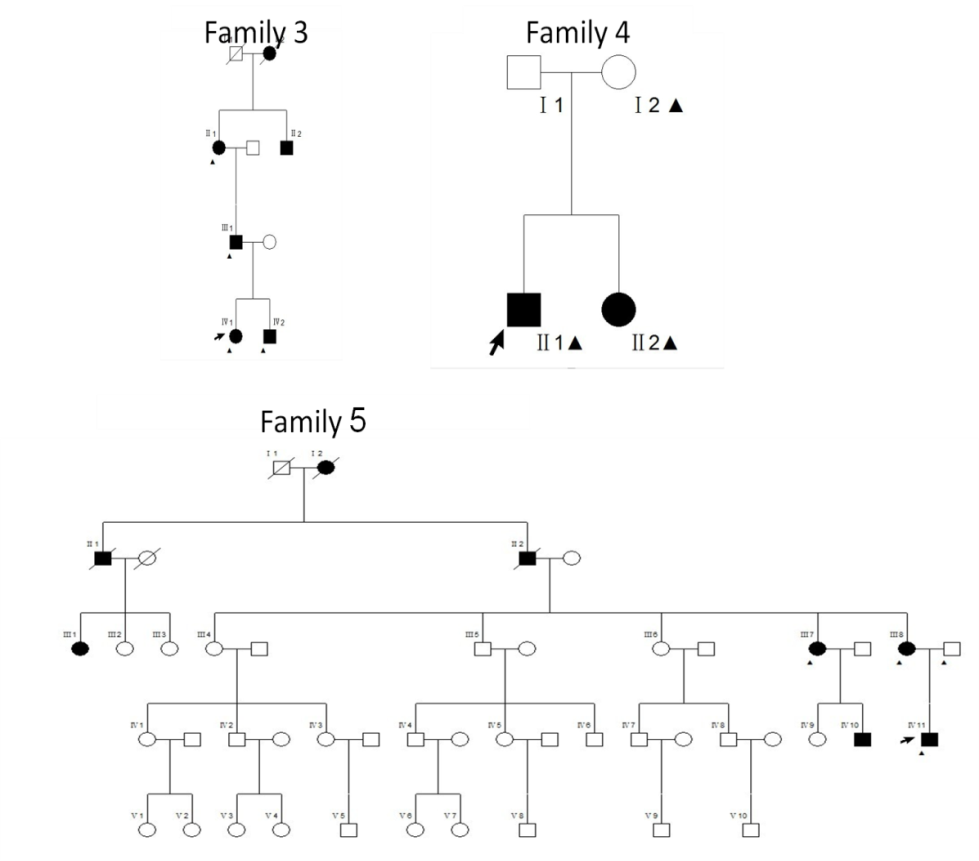
Supplementary Figure 2 : LOD score of parametric linkage analysis vs genetic map for 22 chromosomes

**Supplementary Figure 3: The expression of ABCB6 mRNA in DUH patients and health controls.**

**
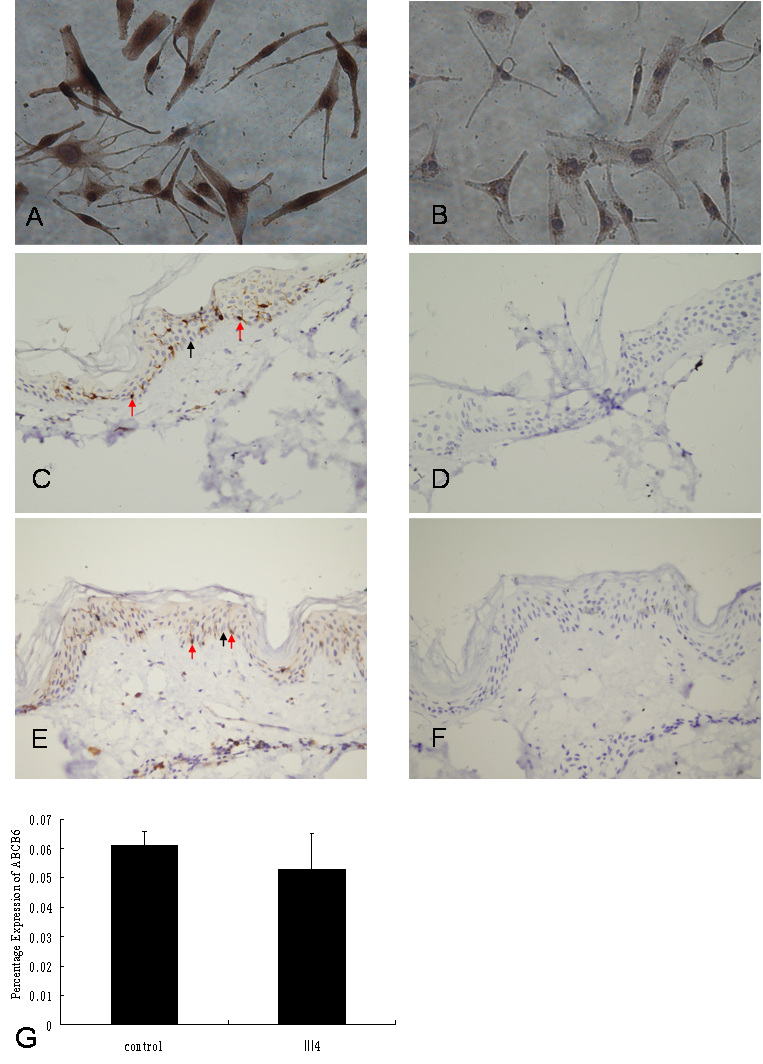
Supplementary Figure 4: The protein level of ABCB6 in normal melanocyte and skin biopsies.** A, B: normal melanocyte; C, D: Skin biopsy sections obtained from Ⅲ4 of family 1 ; E, F: Skin biopsy sections obtained from a health individual; G: The percentage of ABCB6 positive melanocytes was determined in the epidermis (P = 0.27). A, C, E: immunostained with antibody directed against ABCB6 (400 x); B,D,F: negative controls of A, C,E, respectively. Red Arrow: melanocyte; Black Arrow: keratinocyte.

**Supplementary Figure 5. Sequence homology of ABCB6 between species**

**Supplementary Figure 6:** Number of melanocytes in the defined head regions in uninjected control (wildtype), exon 6- and exon 8-morphants and rescued embryos. **P<0.001 (ANOVA one-way analysis of variance with Tukey’s multiple comparison post test).

Supplementary Table 1 Information for exome sequencing reads

|  | **II4 (A)** | **III4 (A)** | **III6 (A)** | **IV4 (A)** |
| --- | --- | --- | --- | --- |
| #Uniquely mapped, paired reads | 73,330,465 | 57,002,192 | 41,747,236 | 116,819,298 |
| #Uniquely mapped, RF,FR reads | 73,082,972 | 56,691,248 | 41,522,948 | 116,109,897 |
| #Mapped bases | 2,277,138,501 | 1.767E+09 | 1.325E+09 | 3.686E+09 |
| Size in ROI | 50,603,526 | 50,700,798 | 50,306,470 | 51,197,688 |
| % bases in ROI | 48.3% | 44.6% | 49.6% | 42.9% |
| Mean coverage | 44.9996014 | 34.854622 | 26.341355 | 71.994064 |
| Coverage StdDev | 44.2749095 | 31.604497 | 25.534448 | 54.907643 |
| % bases covered >=10x | 87.3325 | 86.6363 | 78.9672 | 92.6513 |
